# Current Knowledge and Perspectives of Phage Therapy for Combating Refractory Wound Infections

**DOI:** 10.3390/ijms25105465

**Published:** 2024-05-17

**Authors:** Bo Wang, Lin Du, Baiping Dong, Erwen Kou, Liangzhe Wang, Yuanjie Zhu

**Affiliations:** Department of Dermatology, Naval Medical Center, Naval Medical University, Shanghai 200052, China; wangbo_1993@smmu.edu.cn (B.W.); lynnie_du@126.com (L.D.); dbpjuly@smmu.edu.cn (B.D.); kouew@smmu.edu.cn (E.K.)

**Keywords:** wound infection, wound healing, MDR, antibiotic resistance, phage therapy, alternative treatments

## Abstract

Wound infection is one of the most important factors affecting wound healing, so its effective control is critical to promote the process of wound healing. However, with the increasing prevalence of multi-drug-resistant (MDR) bacterial strains, the prevention and treatment of wound infections are now more challenging, imposing heavy medical and financial burdens on patients. Furthermore, the diminishing effectiveness of conventional antimicrobials and the declining research on new antibiotics necessitate the urgent exploration of alternative treatments for wound infections. Recently, phage therapy has been revitalized as a promising strategy to address the challenges posed by bacterial infections in the era of antibiotic resistance. The use of phage therapy in treating infectious diseases has demonstrated positive results. This review provides an overview of the mechanisms, characteristics, and delivery methods of phage therapy for combating pathogenic bacteria. Then, we focus on the clinical application of various phage therapies in managing refractory wound infections, such as diabetic foot infections, as well as traumatic, surgical, and burn wound infections. Additionally, an analysis of the potential obstacles and challenges of phage therapy in clinical practice is presented, along with corresponding strategies for addressing these issues. This review serves to enhance our understanding of phage therapy and provides innovative avenues for addressing refractory infections in wound healing.

## 1. Introduction

The skin, being the body’s largest organ and constantly exposed to a variety of external threats, plays crucial roles in environmental perception, thermal regulation, physicochemical equilibrium, and protection against physical harm and infections [1,2]. Maintaining these functions relies on the integrity of healthy skin, necessitating not only the prevention of injury but also the efficient process of wound healing [3]. Wound healing is a complex and dynamic process involving multiple phases that work together to restore the integrity of damaged skin through coordinated interactions between growth factors, cytokines, chemokines, and various cells [4,5].

**Figure 1:**
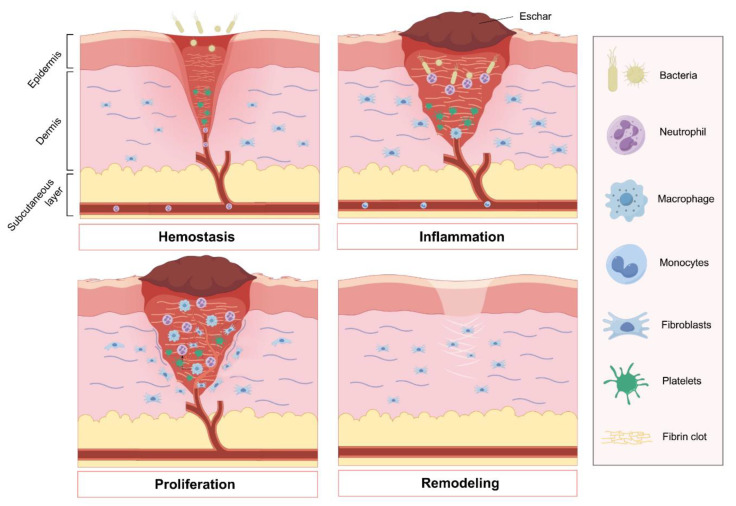
Four phases of wound healing: The initial stage of wound healing, known as hemostasis, involves platelet aggregation to prevent hemorrhage and the formation of an early fibrin clot. Following this, the inflammatory phase is essential for clearing debris and preventing infection, with the recruitment of neutrophils and monocytes that subsequently differentiate into tissue macrophages. The proliferation stage encompasses angiogenesis to restore blood vessels and the replacement of the provisional fibrin clot with granulation tissue by fibroblasts. Ultimately, fibroblasts play a key role in remodeling the extracellular matrix, facilitating the restoration of the skin barrier. Figure made with Figdraw.

As shown in Figure 1, both acute and chronic skin wounds are healed in a process that consists of four successive phases: hemostasis, inflammation, proliferation, and tissue remodeling [6,7,8] (Figure 1). Following injury, damaged blood vessels quickly constrict and form a blood clot to prevent excessive bleeding. Platelets play a vital role in recruiting immune cells to the site of injury, either by directly capturing immune cells in the scab or by releasing chemokine attractants upon degranulation [9]. Subsequently, innate inflammation emerges as the primary defense mechanism against potential infection of the wound. Neutrophils and macrophages are the key immune cells involved in the inflammatory response, influenced by various intrinsic and extrinsic factors [10]. Unregulated inflammation exacerbates tissue damage and impedes the healing process, while inadequate recruitment of immune cells also impedes repair. The proliferative phase of healing involves the coordinated activation of keratinocytes, fibroblasts, macrophages, and endothelial cells to facilitate wound closure, matrix formation, and blood vessel growth. Fibroblasts play a crucial role in replacing the temporary fibrin-rich matrix with a more durable granulation tissue. The remodeling of the extracellular matrix (ECM) commences with the initial deposition of a fibrin clot and culminates over a span of several years with the development of a mature scar rich in type I collagen [11].

These four phases, which are both overlapping and distinct, follow a well-established sequence and occur at specific time points. The complex and intricate interplay among the immune system, keratinocytes, and dermal cells is crucial in the wound healing process. Risk factors, such as immune deficiency, malnutrition, diabetic state, tissue infection, biofilm formation, inadequate blood supply, and abnormal pH level surrounding the wound sites, can impede the wound healing process, leading to chronic or nonhealing wounds [12,13].

Bacteria play a significant role in impeding the wound healing process [14]. It is well established that bacteria are a natural component of the skin microbiota [15]. When the proliferation of bacteria in wounds surpasses the self-regulatory threshold, a wound infection may manifest clinically [16,17,18,19]. These infections not only impede the overall wound healing process but also contribute to the development of chronic or nonhealing wounds, resulting in substantial healthcare and economic burdens on society [8,20,21]. Several studies have reported that a majority of chronic wounds are colonized by bacteria that produce biofilms. Biofilms can act as the diffusion barrier to protect bacterial cells, playing an essential role in the persistence of infection and antimicrobial resistance. The formation of biofilms exacerbates the difficulty of treating wound infections and impedes tissue repair by inducing persistent inflammation at the wound site [13,22]. Consequently, the implementation of efficacious strategies for managing wound infections is imperative to maintain the normal process of wound healing and prevent the development of chronic, nonhealing wounds.

Previous research has identified *Escherichia coli*, *Pseudomonas aeruginosa*, *Klebsiella pneumoniae*, *Enterobacter cloacae*, and *Staphylococcus aureus* as the predominant pathogens responsible for causing wound infections [23]. In traumatic wounds, the most common bacteria identified were *E. coli*, followed by *Acinetobacter species*, *P. aeruginosa*, *K. pneumoniae*, *Enterococcus faecalis*, and *S. aureus*. In surgical wounds, *S. aureus*, particularly methicillin-resistant strains (MRSAs), has been the predominant causative agent in recent years. In diabetic foot ulcers, *S. aureus* is the prevalent isolate, together with others such as *P. aeruginosa*, *Enterococcus* spp., *E. coli*, *Enterobacter* spp., and *K. pneumonia*. In burn wounds, the presence of pathogens such as *P. aeruginosa* together with *Enterobacteriaceae* spp. and all multi-drug-resistant (MDR) or even totally drug-resistant organisms can be deadly.

Antimicrobials, such as antibiotics and antiseptics, have been extensively utilized in clinical settings to reduce bacterial burden and facilitate wound healing [24]. Nevertheless, the indiscriminate application of antibiotics has led to the development and dissemination of bacterial antibiotic resistance on a global scale in recent years. The emergence of antibiotic-resistant and multi-drug-resistant (MDR) bacteria poses a significant challenge to the efficacy of conventional antibiotic therapies for infectious diseases [25]. Furthermore, the presence of bacterial biofilms hinders wound healing by limiting the penetration of antibiotics, thereby exacerbating the challenge of managing wound infections [26]. Given the substantial impact of refractory wound infections on both healthcare costs and patient outcomes, there is a pressing necessity to explore novel therapeutic approaches that can address the current obstacles in wound care posed by antibiotic resistance. Potential strategies, such as phage therapy, have been proposed as alternative treatments for bacterial infections.

## 2. Phage Therapy Acts as a Revitalized Weapon to Eliminate MDR Bacteria and Biofilms

### 2.1. History of Phage Therapy

Bacteriophages (phages), which are viruses capable of infecting and replicating within bacterial cells, were first discovered over a century ago [27,28]. They are widely recognized as the most abundant and ubiquitous organisms on Earth, with significant implications for microbial physiology, population dynamics, evolution, and therapeutic applications [29,30]. Phage therapy represents the use of lytic phages to kill specific host bacteria, and it has been successfully applied in several bacterial diseases since 1917, when Felix d’Herelle first reported the use of phages to treat dysentery disease [31,32]. The emergence of penicillin and other antibiotics led to the neglect and marginalization of phage research globally. However, since the 1980s, the rise of antibiotic resistance has presented a significant obstacle to the efficacy of antibiotics in treating bacterial infections, prompting a resurgence of interest in phage research [33,34]. An increasing number of studies are now exploring the mechanisms, characteristics, and clinical applications of phage therapy [27,35,36]. Phage therapy is now considered a potent weapon for eradicating MDR bacterial strains and combating refractory infections [37].

### 2.2. The Mechanisms and Characteristics of Phage Therapy

**Figure 2:**
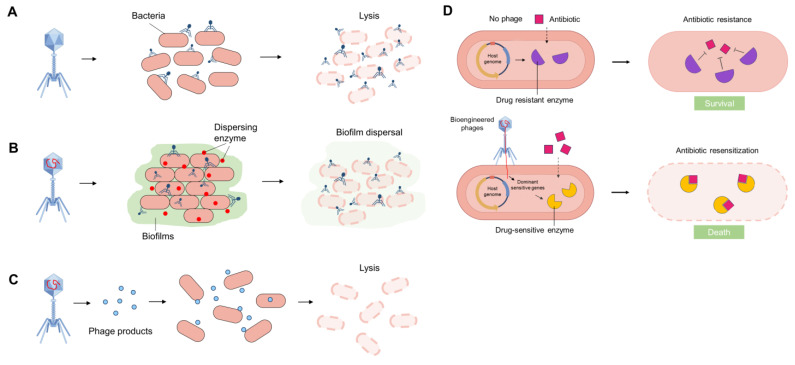
Schematic diagram of phage antimicrobial therapy: Phages and their products provide routes that could lead to the creation of novel antimicrobial strategies. (**A**) Phages can irreversibly recognize and bind to particular bacterial pathogens and induce cell lysis during replication. (**B**) Phages can disrupt biofilms by selectively targeting bacteria within these complex structures, and they can also produce depolymerases, lysozymes, and lytic enzymes to disperse biofilms and induce cell lysis. (**C**) Some phage-derived recombinant products, such as enzymes, peptides, and small molecules, can be used to target specific bacteria and induce cell lysis. (**D**) Phages can sensitize antibiotic-resistant bacteria, and this strategy can be combined with antibiotic treatment. For example, bioengineered phages can introduce dominant antibiotic-sensitive genes into drug-resistant hosts. Subsequently, the expressed drug-sensitive enzyme can bind to antibiotics and reverse/cancel antibiotic resistance. Figure made with Figdraw.

Previous research has demonstrated that phages exert antibacterial effects through four primary mechanisms [38,39,40,41]. Firstly, virulent phages can specifically attach to the surface of pathogenic bacteria and introduce their genetic material into the bacterial host. Subsequently, the phage DNA undergoes replication and utilizes bacterial protein machinery to synthesize structural proteins, which are subsequently assembled into new viral particles within the host cells. This process ultimately results in bacterial lysis and the release of newly formed viruses (Figure 2A).

Secondly, phages demonstrate significant efficacy in penetrating biofilms and inducing bacterial lysis. They can reach the inside of biofilms through the water channel and disrupt biofilms by selectively targeting bacteria within these complex structures. In addition, certain phages can naturally produce various types of depolymerases, lysozymes, and other lytic enzymes, such as hydrolases, lyases, and lipases, which can disperse biofilm matrix and lyse bacteria (Figure 2B). Considerable research has been dedicated to the study of endolysins, peptidoglycan hydrolases that play a crucial role in biofilm degradation and cell lysis. The disruption of structural integrity resulting from peptidoglycan degradation leads to cell lysis due to osmotic imbalance.

Thirdly, phage-derived recombinant products, such as enzymes, peptides, and small molecules, can exhibit enhanced antimicrobial and antibiofilm properties (Figure 2C). For instance, bioengineered phages have been utilized to produce recombinant endolysins fused with cell-penetrating domains, enabling the efficient delivery of these endolysins through the Gram-negative outer membrane or into eukaryotic cells to target intracellular bacteria. Alternatively, some biofilm-degrading enzymes like dispersin B can be produced by bioengineered phages and promote extracellular polymeric substance (EPS) degradation and reduce bacterial biofilm formation.

Lastly, phages can restore the susceptibility of drug-resistant bacteria to antibiotics by several strategies, offering a potential adjunct to conventional antibiotic therapy. For example, bioengineered phages can introduce dominant antibiotic-sensitive genes into drug-resistant hosts. Subsequently, the expressed drug-sensitive enzyme can bind to antibiotics and reverse/cancel antibiotic resistance (Figure 2D). The approach of phage therapy primarily involves the use of lytic phages that selectively target bacterial cells, leaving human cells intact and minimizing disruption to commensal bacteria. This evolving therapeutic strategy has shown promising outcomes in both life-saving interventions and ongoing clinical investigations.

In addition to the aforementioned descriptions, phages demonstrate various advantages and characteristics as an antimicrobial agent for adjunctive therapy in the treatment of MDR bacterial infections, including (1) clinical safety, being relatively free of side effects; (2) easy and rapid isolation with lower development costs; (3) rapid distribution throughout the body with an increased concentration at the site of infection; (4) reduced collateral damage to the microbial community; (5) absence of cross-resistance to antibiotics; (6) potential reversion of bacterial antibiotic susceptibility in vivo; (7) biofilm degrading activity; (8) anticipated cost-effectiveness of pharmaceutical development; and (9) amenability to engineering [42,43,44,45,46,47,48].

### 2.3. Phage Delivery Systems to Infectious Wounds

**Figure 3:**
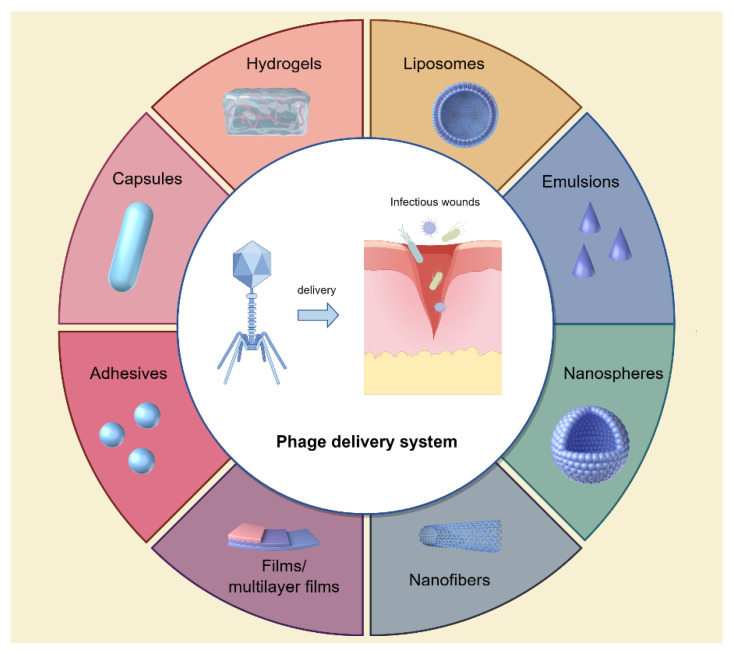
Novel phage delivery systems to infectious wounds. Figure made with Figdraw.

In addition to the application of bacteria-sensitive phages, optimal phage delivery systems are also critical to achieve better antibacterial efficacy. Previously, phages were often used within topical solutions, including ointments, creams, and lotions, or administered in their free form. These formulations are convenient to apply and remove, exhibit stability during treatment, and demonstrate minimal toxicity to patients while facilitating wound healing. However, these methodologies have certain drawbacks [49,50]. The movement of phages to the desired wound sites may be limited by the components of thicker ointment, while the preservative agents in creams and ointments could potentially diminish the efficacy of phages [51]. In order to address these issues, various biomaterials such as capsules, hydrogels, films/multilayer films, nanofibers, and emulsions have been utilized to encapsulate phages, facilitating the transportation of viable phages to the desired infectious wound sites [52,53] (Figure 3).

Hydrogel, a three-dimensional network of hydrophilic polymers, can swell in water and hold a large amount of water while preserving its structural integrity. It has garnered considerable attention as a material for wound dressings and drug depots for prolonged drug release [54]. Recently, hydrogels have been engineered to incorporate phages for the treatment of wound infections [55,56]. Yan et al. conducted a study to evaluate the effectiveness of a phage-loaded thermosensitive hydrogel in addressing wound infections induced by MDR *A. baumannii* using pig skin. In this study, the IME-AB2 phage and MDR-AB2 were utilized as the model phage and bacteria, respectively. The researchers investigated the fundamental characteristics of IME-AB2 phage-hydrogel and assessed its antibacterial effectiveness against MDR-AB2 through in vitro and ex vivo experiments on pig skin. The results demonstrate that phages incorporated in a Poloxamer 407 (P407) hydrogel solution displayed remarkable stability, with a sustained release profile resulting in a cumulative release of 60% within the initial 24 h period [57]. The results of in vitro and ex vivo experiments indicate that the incorporation of phage into the hydrogel had minimal effects on the bactericidal efficacy of the phage, while also serving as a reservoir to sustain elevated phage concentrations at the infection site over an extended period, thereby enhancing treatment efficacy. These findings highlight the potential of a P407-based phage-loaded thermosensitive hydrogel as a straightforward and effective formulation for addressing wound infections, particularly those induced by MDR *A. baumannii*.

Additionally, liposomes, being biocompatible, biodegradable, and nonimmunogenic, have been employed for phage encapsulation. These phage-loaded liposomes function as a depot at the wound sites, facilitating the release of phages at high concentrations [58,59]. Chhibber et al. conducted a study to assess the efficacy of phage cocktail-loaded liposomes in treating *S. aureus*-induced diabetic excision wound infection [58]. In this study, two characterized *S. aureus*-specific lytic phages, MR-5 and MR-10, in combination (cocktail) were entrapped in liposomes, and researchers investigated the efficacy of phage-cocktail-loaded liposomes in treating wound infections in a diabetic mouse model infected with *S. aureus*. The findings indicated that encapsulating the phage cocktail in liposomes led to a higher concentration of viable phages at the wound sites, resulting in accelerated wound healing compared with a free, nonencapsulated phage cocktail. The entrapment of phage cocktails within liposomes may present a promising approach for the treatment of *S. aureus* infections, especially in diabetic wound infections.

In another study, Chadha et al. entrapped a phage cocktail by mixing five different purified phage preparations (KØ1, KØ2, KØ3, KØ4, and KØ5) in liposomes and evaluated its therapeutic efficacy in resolving burn wound infections caused by *K. pneumoniae* B5055 in a mouse model [59]. The findings indicated that mice administered with a liposomal-encapsulated phage cocktail exhibited a greater decrease in bacterial burden in both the bloodstream and vital organs. This was concomitant with a more rapid resolution of the infection compared with those treated with a nonliposomal, free phage cocktail. Additionally, the liposomal phage formulation demonstrated the ability to prevent mortality in all experimental subjects, even when therapy was delayed by 24 h. These findings demonstrated the promise of utilizing liposome-encapsulated phage cocktails as a treatment for infections caused by *K. pneumoniae*. This approach may offer an effective alternative for managing burn wound infections caused by *K. pneumoniae* in patients who are unresponsive to traditional antibiotic treatments.

Other novel biomaterials, such as nanofibers/nanospheres, emulsions, fibrin glues, adhesives, and films, have also been utilized for the encapsulation and delivery of phages to infectious wounds [53,60,61]. Esteban et al. incorporated phage-K into nanoemulsions and evaluated its stability and efficacy in the treatment of *S. aureus* infections [60]. Their findings indicated that the emulsions containing phages exhibited enhanced stability and demonstrated the ability to induce rapid and complete bacterial eradication of three distinct strains of *S. aureus* in comparison with phages that were freely suspended. Additionally, Rubalskii et al. integrated phage PA5 into fibrin glues, monitoring the phage release from fibrin scaffolds and assessing the antibacterial efficacy of the released phages [61]. Their study revealed that the PA5 phages delivered via fibrin glues exhibited high titers over an 11-day incubation period in a liquid medium, effectively targeting and eliminating *P. aeruginosa* PA01. These results suggest that fibrin glues have the potential to serve as a sustained delivery system for phages, offering a promising approach for various antibacterial interventions.

While not yet implemented in clinical settings, the findings unequivocally illustrate the capacity of emerging delivery systems to effectively release phages with high titers at wound sites, resulting in a significant reduction in bacterial colonization and inflammation in infected wounds. Furthermore, phages incorporated into novel delivery systems demonstrate enhanced stability and biocompatibility compared with conventional delivery materials or free phages, indicating promising prospects for their future clinical application in treating wound infections.

## 3. Clinical Application of Phage Therapy to Treat Refractory Wound Infections

In the early years, phage therapy was mainly used to treat severe systemic multi-drug-resistant infections, especially for patients who did not respond to traditional antibiotic treatments. In recent years, with the continuous development of phage therapy and more abundant phages being applied in clinical practice, phage therapy has been widely used to treat a variety of refractory wound infections, including traumatic, surgical, and burn wound infections, as well as diabetic foot infections.

### 3.1. Traumatic Wound Infections

Traumatic wounds present a significant challenge to surgeons due to their intricate nature, extensive size, and high levels of contamination and infection [62]. Prior studies have confirmed that nearly all traumatic wounds exhibit some degree of contamination [63], and the common bacteria identified in such wounds are *E. coli*, *Acinetobacter species*, *P. aeruginosa*, *K. pneumoniae*, *Enterococcus faecalis*, and *S. aureus* [64,65]. It is important to note that bacteria found in the wound may originate not only from the patient’s own skin microflora or external sources during trauma but also from the hands of the examiner [66]. Moreover, recent data indicate that a significant proportion of traumatic wounds are afflicted with polymicrobial infections, with biofilm formation commonly observed at the wound sites. This phenomenon often leads to resistance against traditional antibiotic treatments [67,68]. Failure to effectively address these infections can result in the development of refractory infections, leading to progressive tissue necrosis, septicemia, organ failure, and potentially fatal outcomes.

Phage therapy has significantly impacted the treatment of challenging traumatic wound infections. Bhartiya et al. conducted a nonrandomized prospective unbiased, open-blinded, case-control study to evaluate the effectiveness of phage therapy compared with conventional therapies on large traumatic wounds [69]. The study included 54 patients with wounds larger than 36 cm^2^, who were randomly assigned to receive either conventional antibiotic therapy (CT) or bacteriophage (phage) therapy (BT). Researchers discovered that infectious wounds in the BT group achieved sterility at a significantly faster rate compared with the CT group, with a higher proportion of sterile wound healing observed in the BT group. Moreover, patients in the BT group had a shorter hospital stay and incurred lower expenditures compared with those in the CT group. These findings suggest that phage therapy may be a more effective treatment option for large and refractory traumatic wound infections when compared with conventional wound care methods.

Additionally, Racenis et al. reported a case involving a 21-year-old patient who experienced relapsing multi-drug-resistant *P. aeruginosa* and carbapenem-resistant *A. baumannii* infections following a road accident [70]. Despite receiving extensive antimicrobial therapy and undergoing multiple surgical procedures including wound debridement, the infections persisted. To address the refractory nature of the traumatic wound infection, researchers integrated traditional treatments with the localized administration of a *Pseudomonas* phage cocktail BFC 1.10. Subsequent to the intervention, the patient exhibited no clinical, radiological, or laboratory evidence of inflammation after a period of nine months. The study highlights the potential efficacy of combining phages and antibiotics in the treatment of challenging bone and soft tissue infections.

### 3.2. Surgical Wound Infections

Surgical wound infections, which occur in the vicinity of a surgical incision within 30 or 90 days of surgery, are among the most prevalent hospital-acquired infections [71,72]. Endogenous pathogens are primarily responsible for the development of surgical wound infections, with *S. aureus*, particularly methicillin-resistant strains (MRSAs), being the predominant causative agent in recent years [11]. In addition, patients with specific medical conditions such as cancer and diabetes are at a heightened risk for developing MDR bacterial infections, such as *P. aeruginosa*, *E. coli*, *Streptococcus* spp., and *K. pneumoniae*. These infections can greatly diminish the effectiveness of traditional antibiotic treatments [73]. Apart from bacterial infections and antibiotic resistance, the presence of biofilm poses another challenge in the management of surgical wound infections. Despite advancements in infection prevention measures, surgical wound infection remains a significant contributor to postoperative complications, extended hospital stays, elevated healthcare expenses, and potential mortality. Phage therapy may provide innovative direction to address these challenges.

Notably, phages have been effectively utilized in the treatment of postoperative infected wounds since the early 1940s and 1980s [74,75]. Recent studies have demonstrated the potential of phage therapy in managing surgical wound infections in both human patients and animals, as well as in eradicating biofilm and combating antibiotic-resistant infections in surgical wounds [76]. Leitner et al. conducted a clinical trial in which 474 patients undergoing transurethral resection of the prostate were administered a combination of phage and antibiotic therapy [77]. Prior to treatment, all patients exhibited positive urine cultures for *Streptococcus* spp., *Proteus mirabilis*, *E. coli*, and *P. aeruginosa*. Following seven days of phage therapy, the majority of patients showed a reduction in urinary tract infection symptoms, with fewer adverse effects compared with the antibiotic group, demonstrating the effectiveness and safety of phage therapy.

Additionally, Nadareishvili et al. performed a retrospective analysis on four surgical patients treated at a medical center, including two with chronic osteomyelitis, one with a diabetic foot ulcer, and one with a severe infectious complication following skin grafting surgery [78]. All patients suffered from MDR bacterial infections and were treated with various combinations of phage preparations. The study found that phage therapies had beneficial effects in all cases, improving the patients’ overall health and facilitating wound healing. A case report detailed the management of a recurring knee periprosthetic joint infection with MDR *P. aeruginosa* in an 80-year-old woman [79]. Phage therapy was utilized in conjunction with an antibiotic-loaded cement spacer at the surgical site, resulting in the absence of bacterial isolation from drainage fluids and the rapid recovery of surgical wounds. These findings, along with the above clinical trial and retrospective analysis, provide substantial evidence supporting the efficacy of phage therapy, either alone or in combination with antibiotics, as a novel and promising strategy for addressing challenging surgical infections.

### 3.3. Burn Wound Infections

Severe burn injuries are the most traumatic and physically debilitating injuries affecting multiple organ systems. They can result in considerable morbidity and mortality [80]. Burn patients commonly experience compromised skin barriers, which increase susceptibility to colonization and invasion by pathogenic bacteria, leading to burn wound infections [81]. Pathogen presence in burn wounds varies across different stages of healing, with initial colonization primarily by Gram-positive bacteria, including commensal skin *staphylococci*, in the early days post injury [82]. After a few days, Gram-negative bacteria, specifically *P. aeruginosa*, originating from the patient’s gastrointestinal tract and/or hospital environment, often become prevalent pathogens in burn wound infections [83]. In recent years, the treatment of burn wound infections has become more challenging due to the rise of MDR or pan-drug-resistant (PDR) organisms, leading to severe systemic complications such as systemic infection, systemic inflammatory response, and sepsis [84,85]. Phages show promise as a treatment for burn wound infections, potentially preventing the progression to sepsis and septic shock [86].

Doudi et al. conducted a study aimed at isolating and characterizing phages that are effective against highly drug-resistant bacteria isolated from burn wound samples of 50 patients [87]. The findings revealed that the extensively drug-resistant (XDR) *A. baumannii* strain, a predominant antibiotic-resistant pathogen in specialized burn units, exhibited notable susceptibility to phage PΦ-Bw-Ab. This phage can be a suitable candidate to treat burn wound infections, suggesting that phage therapy could serve as a viable alternative to antibiotics, particularly in instances of antibiotic resistance.

Furthermore, Fralick et al. conducted a study to evaluate the effectiveness of phage therapy in treating *P. aeruginosa* infection in a mouse burn wound model [88]. Their findings revealed that a single dose of the *P. aeruginosa* phage cocktail led to a notable reduction in mortality among burn-injured mice infected with *P. aeruginosa*. Additionally, the route of administration played a crucial role in the treatment’s efficacy, with the intraperitoneal route demonstrating the highest level of protection at 87%. The emergence of antibiotic-resistant *P. aeruginosa* as a significant pathogen in burn wound infections has been associated with high mortality and morbidity rates in severe cases [89]. Recent research has highlighted the potential efficacy of phage therapy in addressing multi-drug-resistant bacterial infections in burn wounds, offering valuable insights into the optimal routes of administration for clinical treatment.

### 3.4. Diabetic Foot Infections

Diabetic foot infections (DFIs), stemming from diabetic foot ulcers, have emerged as a significant public health issue. The progression of DFIs may result in amputation in around 20% of moderate to severe cases [90,91]. A variety of microorganisms can be identified in DFIs, with *S. aureus* being the predominant isolate, alongside *P. aeruginosa*, *Enterococcus* spp., *E. coli*, *Enterobacter* spp., *Proteus mirabilis*, and *K. pneumonia* [92,93,94]. Antibiotic resistance in DFIs is common, as evidenced by the high prevalence of methicillin-resistant *S. aureus* in DFIs, estimated to be between 15–30% [95]. Given the significant clinical and financial challenges associated with diabetic foot infections, it is crucial to investigate alternative treatment approaches.

Multiple studies have demonstrated the efficacy of phage therapy in the treatment of DFIs. Jones et al. conducted a study in which they administered topical adjunctive antistaphylococcal phage therapy to 10 DFI patients at high risk of amputation [96]. The study evaluated the tolerability and efficacy of specific phage therapy in treating DFIs. Results show that the majority of bacterial isolates from DFIs were susceptible to the phage therapy, leading to nearly complete clinical resolution of the infections. This highlights the significant superiority of phage therapy in treating refractory DFIs compared with conventional therapies.

Additionally, Mo et al. conducted a study on 185 DFI samples, which revealed that two phages isolated from sewage samples displayed a broad antibacterial effect against *P. aeruginosa* isolated from DFIs. These phages also maintained heat and pH stability over a period of 1 h [97]. Both *S. aureus* and *P. aeruginosa* are significant opportunistic multi-drug-resistant bacteria in diabetic foot infections, impeding the healing of diabetic foot ulcers (DFUs). These findings significantly demonstrate the unique value and promising potential of phage therapy in treating refractory DFIs and promoting the healing of DFUs.

Note: Several significant studies on phage therapy against refractory wound infections are shown in Table 1.

## 4. Perspectives and Challenges of Phage Therapy

The emergence of multi-drug-resistant bacteria and the diminishing focus on antibiotics research have led to a growing interest in phage therapy as a potential alternative treatment. Despite its promising outlook, there are several issues and challenges that must be resolved before widespread clinical implementation. One such challenge is the specificity of phages for their hosts, which presents both advantages and disadvantages for phage therapy [104,105]. High specificity means that the precise diagnosis and identification of infectious bacteria is the prerequisite for the successful implementation of phage therapy. This process can be challenging, time-consuming, and resource-intensive [105]. Moreover, most of the wounds are co-infected with multiple bacteria [106] or diverse subtypes of a single pathogenic bacteria, so the high host specificity of phages may greatly limit the scope of their application. This limitation represents a significant barrier to the widespread application of phage therapy on a large scale.

**Figure 4:**
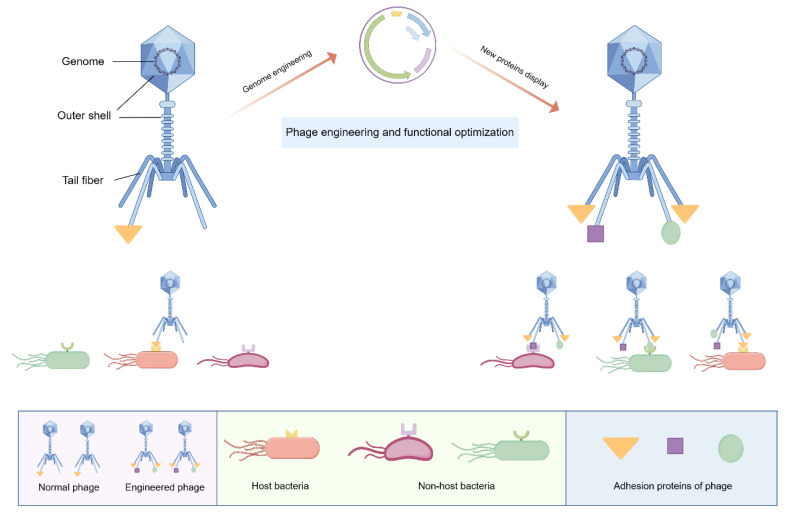
Schematic diagram of phage engineering: Phages have traditionally been characterized as exhibiting high specificity towards their host bacteria, primarily attributed to the specific interactions between adhesion proteins and their corresponding receptors. Phages consist of an outer shell and an internal genome. Through genetic manipulation, phages can be engineered to express specific adhesion proteins in their tail fibers. These modified phages are capable of binding to a wider range of pathogenic bacteria and demonstrating a broader spectrum of antibacterial activity. Figure made with Figdraw.

Phage engineering presents a potential solution to this problem, as indicated by previous research demonstrating the structural simplicity of phages, consisting of a core and protein capsid, which facilitates their modification for enhanced functionality [107]. The adhesion proteins expressed in tail fibers are essential for the invasion ability of lytic phages. As shown in Figure 4, adhesion proteins have the capability to attach to distinct receptors located on the exterior of the host bacteria and exert high specificity [104]. Through the modification of receptor-binding proteins and the structural domains within phages, it is possible to redirect their specificity towards pathogenic bacteria, thereby broadening the antibacterial spectrum and enhancing the practical utility of phages. Likewise, the optimization of additional characteristics of phages through genome engineering can also enhance their antibacterial efficacy, warranting further investigation in future studies.

Secondly, the emergence of bacterial resistance during phage treatment is also a significant concern, particularly following prolonged use of phages [108,109]. Certain studies have raised concerns about phage therapy against biofilm-related infections, as rapid development of phage resistance has been observed [110]. Combining phage therapy with antibiotics may offer a potential solution to this issue. Several studies have demonstrated that the evolution of bacteria towards phage resistance can result in heightened susceptibility to antibiotics, thereby diminishing bacterial pathogenicity and facilitating the management of infections [111,112]. For example, Chan et al. reported a case of antibiotic resensitization wherein the receptor-binding site for phage OMKO1 was found to overlap with two multidrug efflux systems, and the development of phage resistance led to the inactivation of these efflux pumps, consequently restoring antibiotic sensitivity [111]. Consequently, there has been a growing interest in utilizing antibiotics as adjuncts to phage therapy in order to promote antibacterial outcomes. Moreover, researchers have undertaken both in vitro and in vivo studies, validating the antibiofilm efficacy of combining phages and antibiotics in refractory wound infections [113,114]. Additionally, the use of a “cocktail” phage therapy, which involves the combination of multiple phages with diverse mechanisms, has shown effectiveness in mitigating phage resistance. Nevertheless, additional research is required to establish standardized protocols for the selection and proportions of phages included in these cocktails.

Thirdly, the parameter of pharmacokinetics/pharmacodynamics (PK/PD) can provide an accurate representation of the temporal dynamics of the antibacterial activity of antimicrobial agents within the body. Suitable dosage regimens based on PK/PD principles have been shown to enhance the effectiveness of antimicrobial treatments and mitigate the development of bacterial resistance. In comparison to internal organ infections, the management of skin wound infections necessitates a greater reliance on PK/PD considerations. Nevertheless, the understanding of PK/PD in the context of phage therapy remains limited in contrast to traditional antibiotic therapies. Further investigation into phage PK/PD is imperative for future therapeutic applications [115].

Finally, despite the abundance of recent studies on phage therapy, further well-designed clinical trials are necessary to establish its efficacy and safety as a novel antimicrobial treatment. This will help address the unfamiliarity and resistance hindering its widespread application in clinical practice. Despite the current challenges, we firmly believe that phage therapy will emerge as a powerful, safe, and reliable weapon against refractory wound infections in the future.

Note: The advantages of phage therapy and the problems that still need to be solved are listed in Table 2.

## 5. Conclusions

Wound healing is an intricate process impacted by various factors, with wound infection serving as a crucial determinant. The increasing prevalence of chronic or nonhealing wounds stemming from refractory infections has placed a substantial burden on both patients and healthcare systems. These infections are particularly difficult to manage due to the presence of biofilm-forming and multi-drug-resistant bacteria. In the post-antibiotic era, phage therapy has regained prominence due to its demonstrated efficacy in addressing a range of persistent wound infections, including traumatic, surgical, and burn wound infections, as well as diabetic foot infections. Researchers have confirmed the bactericidal properties of phages, which are capable of targeting antibiotic-resistant bacteria, thereby presenting a promising alternative to conventional antibiotic treatments. Furthermore, the development of novel phage delivery systems, such as hydrogels, liposomes, nanospheres, emulsions, adhesives, and films, enables the transportation of viable phages to desired sites, enhancing the potential of phage therapy in treating wound infections. However, it is essential for phage therapy to adhere to established guidelines to maintain efficacy and stability during treatment. Further research is needed to establish phage therapy as a standard medical practice in the future.

## Figures and Tables

**Table 1:** Several significant studies of phage therapy against refractory wound infections in human and mouse cases.

Type	Isolated Bacteria	Phage Name	Main Results	Author Name	Reference
Traumatic wound infection	*E. coli*, *P. aeruginosa K. pneumoniae*, *E. faecalis*, and *S. aureus*	Non-specific phage cocktail	The wounds became sterile within 14 days of the customized BT and 81.5% of the wounds on BT could heal by primary intention.	Bhartiya et al.	[69]
	MDR *P. aeruginosa* and carbapenem-resistant *A. baumannii*	Cocktail BFC 1.10	Nine months after the intervention, the patient showed no signs of inflammation based on clinical, radiological, and laboratory assessments.	Karlis et al.	[70]
	Drug-resistant *P. aeruginosa*, *S. aureus*, and *E. coli*	-	A significant improvement was observed in the wound healing, and there were no signs of infection clinically and microbiologically after 3 to 5 doses of topical phage therapy.	Gupta et al.	[98]
Surgical wound infection	*Streptococcus* spp., *P. mirabilis*, *Enterococcus* spp., *E. coli*, *P. aeruginosa*, and *Staphylococcus* spp.	Pyophage	60% of patients exhibited a lack of urinary tract infection symptoms post treatment, along with a decrease in colony-forming units, as observed in microbiological outcomes.	Leitner et al.	[77]
	MDR bacterial infection such as *B. cepacia*, *S. aureus*, and *E. faecalis*	Pyophage and antistaphylococcal phage	Phage therapy demonstrated a beneficial impact on all four cases, leading to an enhancement in overall health status and wound healing.	Nadareishyili et al.	[78]
	MDR *P. aeruginosa*	-	No bacterial isolates were detected in drainage fluids collected on days 3, 4, and 5 following phage treatment.	Tkhilaishvili et al.	[79]
	*P. aeruginosa*	Cocktail of 12 natural lytic phages	The phage therapy was effective on bacterial colonies isolated from wounds.	Jault et al.	[99]
Burn wound infection	XDR *A. baumannii* strain	Phage PΦ-Bw-Ab	XDR *baumannii* strain IAU_FAL101 showed significant sensitivity to phage PΦ-Bw-Ab.	Doudi et al.	[87]
	*P. aeruginosa*	*P. aeruginosa* phage cocktail	Administration of a single dose of the *P. aeruginosa* phage cocktail significantly reduced mortality in burn-injured mice infected with *P. aeruginosa*, with the intraperitoneal route providing the most effective protection.	Fralick et al.	[88]
	MDR *Enterobacter cloacae and E. hormaechei*	Phages PΦEn-CL and PΦEn-HO	*E. cloacae* strain Iau-EC100 and *E. hormaechei* strain Iau-EHO100 isolated from burn wounds were sensitive to the isolated phages. Phages had no significant toxicity effect on human skin cells	Torabi et al.	[100]
	*P. aeruginosa* PAO1 strain	Engineered superinfective Pf phage	Pf phage treatment completely abolished the capability of P. aeruginosa to disseminate from the burn site to internal organs. And over the course of 10 days, this resulted in bacterial clearance and survival of all treated mice.	Prokopczuk et al.	[101]
Diabetic foot infection	*S. aureus* and *S. hominis*	Anti-staphylococcal phage	Most of the bacteria isolates of DFIs were susceptible to the phage, and the DFIs almost achieved clinical resolution with phage therapy.	Jones et al.	[96]
	MDR *P. aeruginosa*	Phage ϕPAE1 and ϕPAE2	Phages ϕPAE1 and ϕPAE2 exhibited broad antibacterial effects against *P. aeruginosa* clinical strains isolated from DFIs.	Mohamed et al.	[97]
	MDR *K. pneumonia*	Phage cocktail (KP1, KP2, KP3, and KP4)	Phage cocktail demonstrated significantly higher antibacterial activity than each single phage without any bacterial regrowth.	Jokar et al.	[102]
	XDR *S. aureus*	*S. aureus* myoviridae phages cocktail AB-SA01,	AB-SA01 treatment decreased the bacterial load with efficacy superior to vancomycin treatment, and no mortality was recorded to be associated with infections.	Kifelew et al.	[103]

**Table 2:** The advantages and current problems of phage therapy for wound infections.

Advantages	Current Problems
Clinical safety: relatively free of side effects; Specificity: does not kill microbial community; Low cost: easy and rapid isolation with lower costs; High efficacy: rapidly distribute throughout the body with an increased concentration at the site of infection; Potential reversion of bacterial antibiotic susceptibility; Biofilm degrading activity; Amenability to engineering.	High specificity: causative bacterium must be identified beforehand, narrow spectrum of action; Emergence of bacterial resistance during phage treatment; Not enough well-designed clinical trials supporting its efficacy and safety in clinical practice; Lack of in vivo PK/PD data and specific regulatory framework.
